# Acute Impacts of Ionizing Radiation Exposure on the Gastrointestinal Tract and Gut Microbiome in Mice

**DOI:** 10.3390/ijms25063339

**Published:** 2024-03-15

**Authors:** Alexandra Jameus, Jessica Dougherty, Ramya Narendrula, Daniela Levert, Manon Valiquette, Jake Pirkkanen, Christine Lalonde, Patrice Bonin, Jeffrey D. Gagnon, Vasu D. Appanna, Sujeenthar Tharmalingam, Christopher Thome

**Affiliations:** 1 School of Natural Sciences, Laurentian University, Sudbury, ON P3E 2C6, Canada; ajameus@laurentian.ca (A.J.); ramnarendrula@nosm.ca (R.N.); jpirkkanen@middlebury.edu (J.P.); chrisnlalonde@gmail.com (C.L.); pbonin1@laurentian.ca (P.B.);; 2 Medical Sciences Division, NOSM University, 935 Ramsey Lake Rd., Sudbury, ON P3E 2C6, Canada; 3 Health Sciences North Research Institute, 56 Walford Road, Sudbury, ON P3E 2H3, Canada

**Keywords:** radiation therapy, acute radiation syndrome, gastrointestinal tract, microbiome, biomarkers

## Abstract

Radiation therapy for abdominopelvic malignancies often results in damage to the gastrointestinal tract (GIT) and permanent changes in bowel function. An overlooked component of the pathophysiology of radiation-induced bowel injury is the role of the gut microbiome. The goal of this research was to identify the impacts of acute radiation exposure on the GIT and gut microbiome. C57BL/6 mice exposed to whole-body X-rays (0.1–3 Gy) were assessed for histological and microbiome changes 48 h post-radiation exposure. Within the ileum, a dose of 3 Gy significantly decreased crypt depth as well as the number of goblet cells, but increased overall goblet cell size. Overall, radiation altered the microbial distribution within each of the main phyla in a dose- and tissue-dependent manner. Within the Firmicutes phylum, high dose irradiation resulted in significant alterations in bacteria from the class Bacilli within the small bowels, and from the class Clostridia in the large bowels. The 3 Gy radiation also significantly increased the abundance of bacterial families from the Bacteroidetes phylum in the colon and feces. Overall, we identified various alterations in microbiome composition following acute radiation exposure, which could potentially lead to novel biomarkers for tracking patient toxicities or could be used as targets for mitigation strategies against radiation damage.

## 1. Introduction

Radiation therapy comprises an integral part of cancer treatment in various aspects of disease management. From local field treatment of the affected site to managing symptoms of advanced disease, roughly half of all patients diagnosed with cancer can benefit from treatment using carefully construed fields of ionizing radiation [1,2]. Unfortunately, there is currently no way to completely avoid the irradiation of healthy tissues, which often leads to patients experiencing reduced quality of life from the associated side effects. Many abdominopelvic structures possess relatively low radiation tolerances, largely due to their rapid cell turnover, which works against the high doses of radiation needed to abolish a tumour in the area. This poses problems when planning treatments. Specific primary tumour sites which have a tendency to result in radiation-induced bowel injury include the prostate, cervix, urinary bladder, testes, uterus, rectum, and anus [2].

The effects of radiation on the gastrointestinal tract (GIT) include a long list of both acute and chronic adverse outcomes that can lead to significant radiation-induced morbidity. The acute effects of radiation manifest clinically in the days following exposure [3]. At the level of the bowels, radiation-induced enteritis develops in roughly 50% of patients [4]. In the small bowel, acute effects primarily manifest in the form of pain and diarrhea [2]. In the rectum, radiation proctitis presents both acutely and chronically in 5–20% of patients [2]. It has been frequently suggested that up to 90% of patients will experience permanent changes in bowel function following treatment and at least half of all patients claim to have their quality of life impacted by chronic GIT morbidities [3,5,6]. The management of radiation-induced bowel injury often requires a multidisciplinary approach, which in severe cases may consist of surgical management associated with a direct mortality rate of 10–22% [2,3]. Along with the direct damage to the GIT tissue, radiation exposure has been shown to impact the composition of the human gut microbiome (HGM) [3].

Recently, microbiomes have come under greater interest for their roles in mediating various aspects of human health and disease [7,8]. The HGM contains at least 100 times the number of genes compared to the human genome [9,10]. Firmicutes and Bacteroidetes are the dominant phyla of the HGM, comprising roughly 80% of bacteria [3,4,11]. In addition to overall health and disease physiology, the HGM has been shown to be a key player in the response to ionizing radiation exposure [4]. Dysbiosis is seen frequently in patients undergoing radiation therapy [12]. Since both the gut and its microbiome provide bidirectional beneficial signals that result in a symbiotic environment for optimal gut health, dysbiosis can cause a cascade of negative effects on human health.

An increasing amount of research has shown that the gut microbiome may correlate to the efficacy of cancer therapies in patients, as well as the side effects they may experience throughout treatment [3]. For example, the incidence of radiation-induced diarrhea has been linked to alterations in microbiome composition [3,12,13]. Studies have shown that not only does radiation cause dysbiosis, which contributes to radiation-induced bowel injury, but pre-existing dysbiosis may also contribute to the development of many adverse effects seen in patients [14,15]. Specifically, the Firmicutes/Bacteroidetes phyla ratio has been continuously linked to overall gut homeostasis as well as various diseases such as obesity, type 1 diabetes, and inflammatory bowel disease [12,13,15,16,17,18,19,20]. Additionally, at the level of the genus, alterations in *Bacteroides*, *Lactobacillus*, *Roseburia, Escherichia*, *Faecalibacterium*, *Prevotella*, and *Clostridium* are common following radiation exposure [15,18,20,21].

Despite reports of radiation-induced changes to the gut microbiome, questions still remain regarding the overall role that the HGM plays in GIT acute radiation syndrome, delineated by the lack of ability of cells to regenerate following radiation exposure [22]. Previous work has focused mainly on broad changes to the HGM, and few studies have investigated beyond the phyla level. In addition, most studies to date have focused on large doses to the GIT (>5 Gy) that would be accumulated across multiple radiotherapy fractions. However, of interest from a clinical perspective are potential acute short-term microbial changes that could occur as early as a single radiotherapy fraction and could be used as biomarkers of future GIT damage. In addition, during radiotherapy, some of the surrounding GIT tissue will be subject to lower doses (<1 Gy) and while these doses may not directly induce tissue damage, they could still impact the microbial balance in the GIT.

The goal of this study was to investigate short-term radiation effects on the GIT and gut microbiome. C57BL/6 mice were administered a range of X-ray doses (0.1–3 Gy) constituting both low- and high-dose exposures. Radiation effects were quantified 48 h post exposure through histology and 16S metagenomic sequencing of luminal contents and feces. Overall, we identified multiple novel radiation-induced alterations in the relative abundance of bacteria across various taxonomic ranks, including potential microbial-based biomarker targets for the acute monitoring of GIT damage in radiotherapy patients.

## 2. Results

### 2.1. The Effects of Radiation on GIT Histology

**Figure 1:**
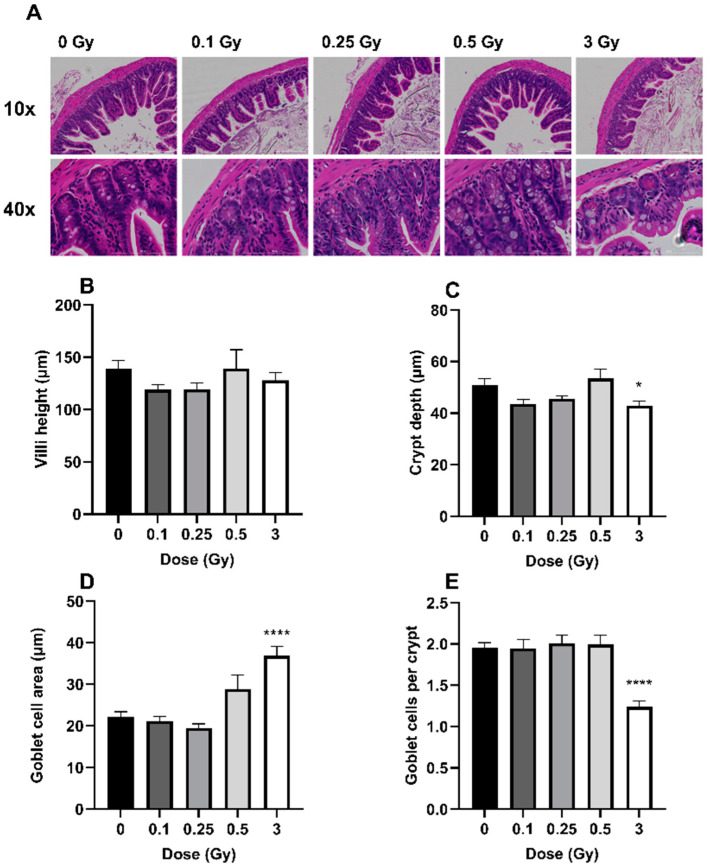
Representative H&E-stained sections of the ileum at each dose at 10× and 40× magnification (**A**). Villi height (**B**), crypt depth (**C**), goblet cell area (**D**), and quantity of goblet cells (**E**) are shown at 48 h post exposure. An average of 24 villi and 103 crypts were scored per mouse. Bars represent the mean ± SEM across replicate animals (*n* = 5–12 per treatment). Data were analyzed using a one-way ANOVA followed by Tukey’s post hoc test. Symbols indicate statistical significance compared to 0 Gy control: * *p* ≤ 0.05, **** *p* ≤ 0.0001.

Histological changes of the ileum were quantified on H&E-stained tissue sections (Figure 1A). Irradiation had no significant impact on ileal villi height (Figure 1B, *p* = 0.36). However, the 3 Gy exposure significantly reduced crypt depth (Figure 1C, *p* = 0.027). In addition, the 3 Gy exposure significantly increased goblet cell area (Figure 1D, *p* ≤ 0.0001) but decreased the overall quantity of goblet cells (Figure 1E, *p* ≤ 0.0001). Compared to sham-irradiated mice, there was a 16% decrease in crypt depth, a 66% increase in goblet cell size, and a 37% decrease in the number of goblet cells following 3 Gy exposure. Crypt depth as well as the overall number and size of the goblet cells were not impacted by the three lower doses.

### 2.2. The Effects of Radiation on the GIT Microbiome

Changes to gut microbiome composition were characterized via a 16S metagenomic analysis. Based on the Shannon index alpha diversity results, the largest bacterial diversity was observed in the colon and the feces, with the lowest diversity in the jejunum and ileum (Supplementary Figure S1). Radiation did not significantly alter the overall alpha diversity in any of the GIT segments (*p* = 0.0674). To examine, in more detail, the effects of radiation on specific bacterial populations, the relative microbial abundance was quantified across various taxonomic ranks.

#### 2.2.1. Radiation Increased the Abundance of Proteobacteria and Deferribacterota in the GIT

**Figure 2:**
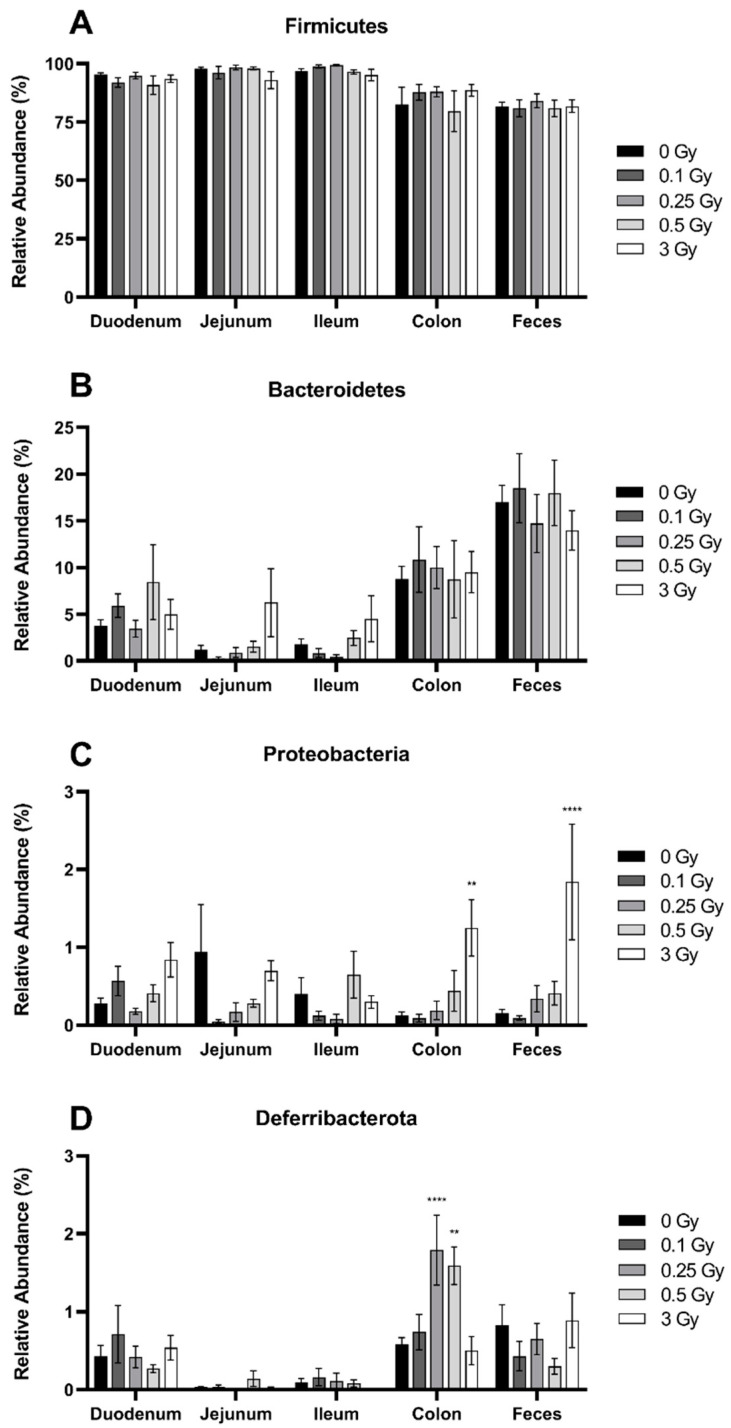
Relative abundance of the bacteria phyla Firmicutes (**A**), Bacteroidetes (**B**), Proteobacteria (**C**), and Deferribacterota (**D**) in the GIT and feces of irradiated mice. Relative bacterial abundance (% of total) was quantified using 16S metagenomics sequencing 48 h post radiation exposure. Bars represent the mean ± SEM across replicate animals (*n* = 4–12 per treatment). Data were analyzed using a two-way ANOVA followed by Tukey’s post hoc test. Symbols indicate statistical significance compared to 0 Gy control within each GIT segment: ** *p* ≤ 0.01, **** *p* ≤ 0.0001.

The Firmicutes was the most dominant phylum within the GIT, comprising roughly 80–99% of the microbiome depending on dose and region (Figure 2A). The relative abundance of Firmicutes was significantly lower in the more distal regions of the GIT (*p* ≤ 0.0001). The second most abundant phylum was Bacteroidetes (Figure 2B). In contrast to Firmicutes, the relative abundance of Bacteroidetes increased towards the distal region of the GIT (*p* ≤ 0.0001), and the highest quantity was located in the feces, comprising 15–18% of the microbiome. Proteobacteria (Figure 2C) and Deferribacterota (Figure 2D) were present across all GIT regions, but each represented less than 1% abundance in sham-irradiated mice.

Exposure to ionizing radiation did not significantly alter the relative abundance of Firmicutes (*p* = 0.59) or Bacteroidetes (*p* = 0.50). However, a dose of 3 Gy significantly increased Proteobacteria in the colon and feces, where the relative abundance was 10.1- and 11.7-fold higher, respectively, compared to sham-irradiated mice (Figure 2C). In the case of Deferribacterota, doses of 0.25 and 0.5 Gy significantly increased the relative abundance 3.1- and 2.7-fold, respectively, in the colon (Figure 2D).

#### 2.2.2. Radiation Altered the Distribution of Firmicutes in the GIT

**Figure 3:**
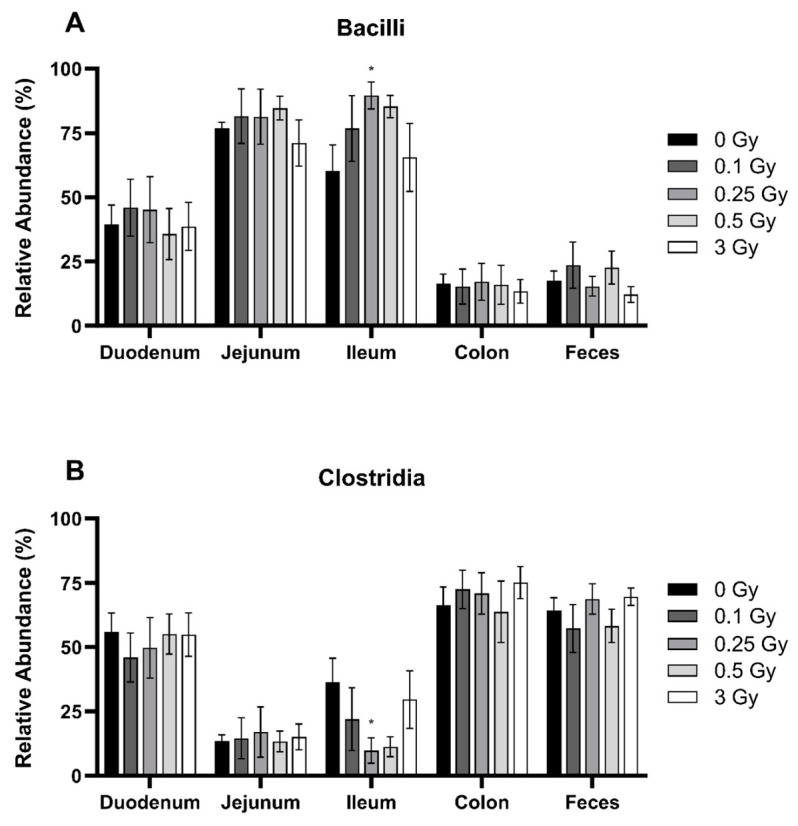
Relative abundance of the bacteria class Bacilli (**A**) and Clostridia (**B**) of the Firmicutes phyla in the GIT and feces of irradiated mice. Relative bacterial abundance (% of total) was quantified using 16S metagenomics sequencing 48 h post radiation exposure. Bars represent the mean ± SEM across replicate animals (*n* = 5–12 per treatment). Data were analyzed using a two-way ANOVA followed by Tukey’s post hoc test. Symbols indicate statistical significance compared to 0 Gy control within each GIT segment: * *p* ≤ 0.05.

Despite the fact that radiation did not significantly impact the abundance of Firmicutes across the GIT segments, it may have still altered the bacterial distribution within the phyla itself. The two main classes of Firmicutes are Bacilli (Figure 3A) and Clostridia (Figure 3B). Bacilli was significantly more abundant in the proximal regions of the GIT (*p* ≤ 0.0001), whereas Clostridia was significantly richer in the colon and feces (*p* ≤ 0.0001). Radiation exposure modestly impacted both classes but only in the ileum. Here, the relative abundance of Bacilli was significantly increased by 1.5-fold following a dose of 0.25 Gy, matched by a decrease in Clostridia of 3.7-fold at the same dose.

**Figure 4:**
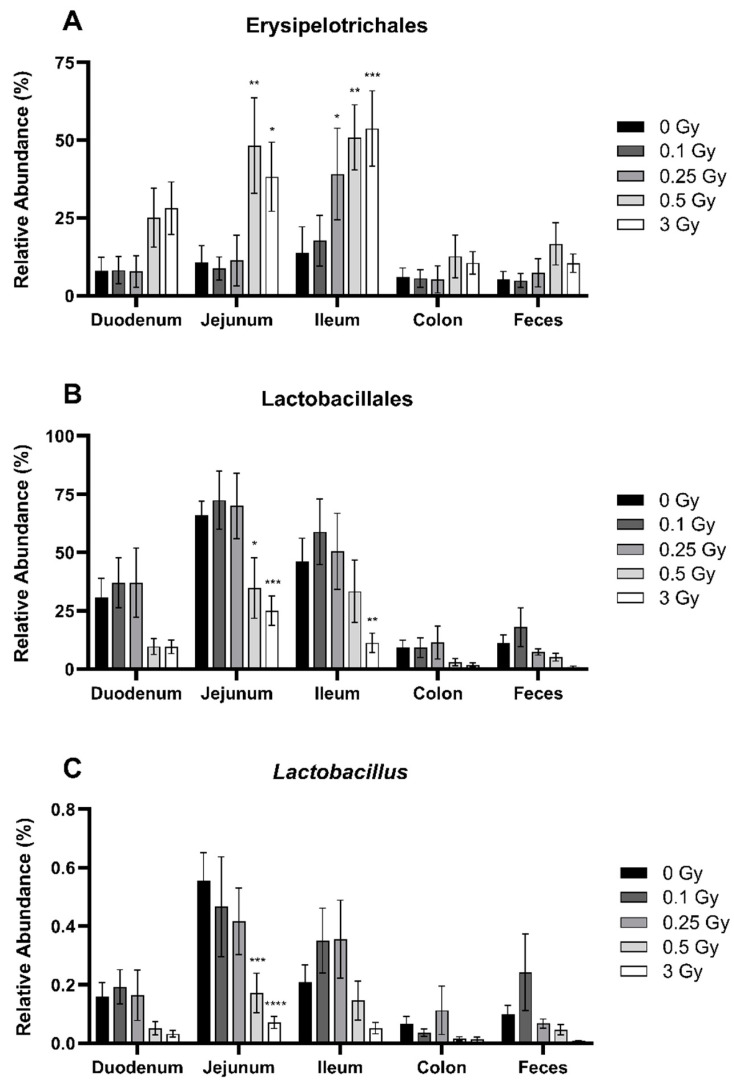
Relative abundance of the bacteria order Erysipelotrichales (**A**), Lactobacillales (**B**), and genus *Lactobacillus* (**C**) in the GIT and feces of irradiated mice. These microbes belong to the bacterial class Bacilli, under the phyla Firmicutes. Relative bacterial abundance (% of total) was quantified using 16S metagenomics sequencing 48 h post radiation exposure. Bars represent the mean ± SEM across replicate animals (*n* = 5–12 per treatment). Data were analyzed using a two-way ANOVA followed by Tukey’s post hoc test. Symbols indicate statistical significance compared to 0 Gy control within each GIT segment: * *p* ≤ 0.05, ** *p* ≤ 0.01, *** *p* ≤ 0.001, **** *p* ≤ 0.0001.

Descending through taxonomic ranks of the class Bacilli, the bacterial orders Erysipelotrichales (Figure 4A) and Lactobacillales (Figure 4B) were analyzed. Ionizing radiation significantly impacted the relative abundance of both orders. Irradiation increased the abundance of Erysipelotrichales in the jejunum (0.5 and 3 Gy) and the ileum (0.25, 0.5, and 3 Gy) by approximately 3–4-fold (Figure 4A). In contrast, high-dose irradiation decreased the abundance of Lactobacillales in the jejunum (0.5 and 3 Gy) and the ileum (3 Gy) by 2–4-fold (Figure 4B).

Looking further within the order Lactobacillales, ionizing radiation significantly impacted the genus *Lactobacillus*. Within the jejunum, doses of 0.5 and 3 Gy significantly decreased *Lactobacillus* abundance by 3.2- and 7.8-fold, respectively (Figure 4C).

**Figure 5:**
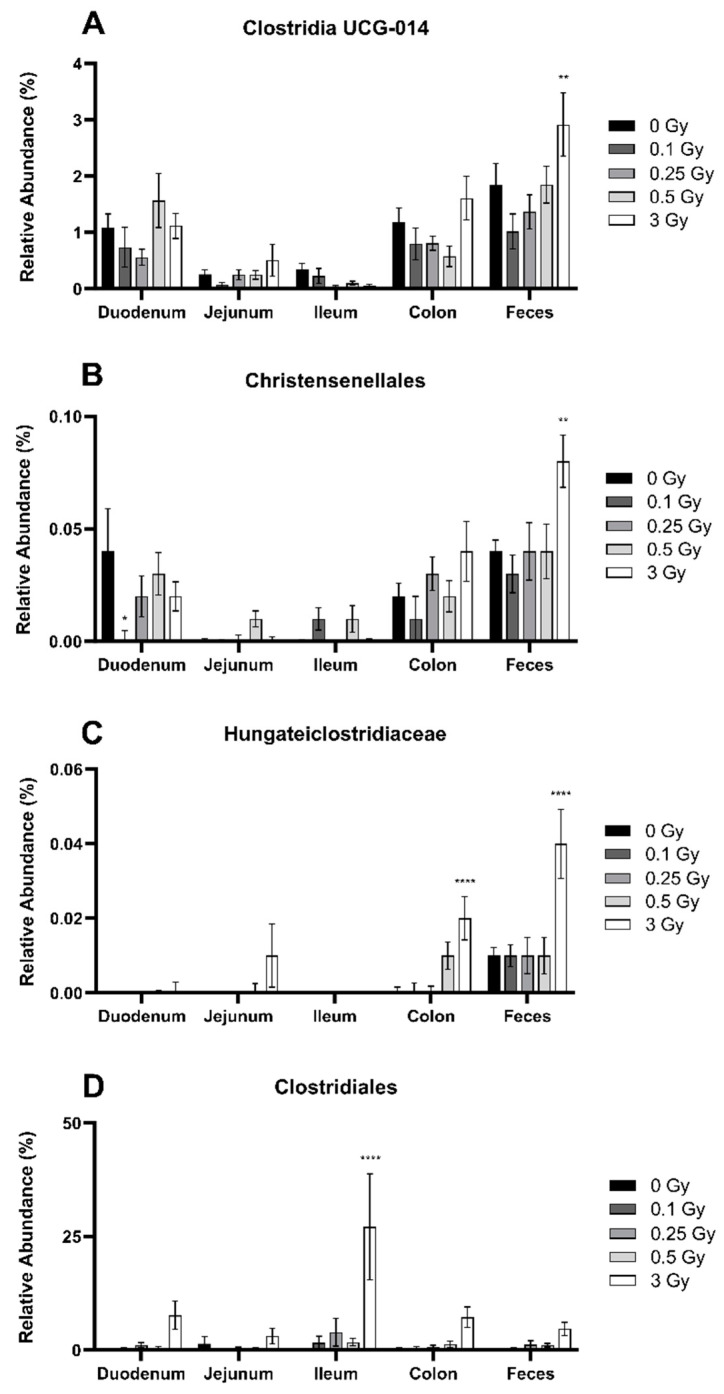
Relative abundance of the bacteria order Clostridia UCG-014 (**A**), Christensenellales (**B**), Hungateiclostridiaceae (**C**), and Clostridiales (**D**) in the GIT and feces of irradiated mice. These microbes belong to the bacterial class Clostridia, under the phyla Firmicutes. Relative bacterial abundance (% of total) was quantified using 16S metagenomics sequencing 48 h post radiation exposure. Bars represent the mean ± SEM across replicate animals (*n* = 5–12 per treatment). Data were analyzed using a two-way ANOVA followed by Tukey’s post hoc test. Symbols indicate statistical significance compared to 0 Gy control within each GIT segment: * *p* ≤ 0.05, ** *p* ≤ 0.01, **** *p* ≤ 0.0001.

Next, the class Clostridia was analyzed in more depth. In contrast to Bacilli, where radiation effects were concentrated within the small intestine, Clostridia was primarily impacted in the distal GIT. Within the feces, the orders Clostridia UCG-014 (Figure 5A), Christensenellales (Figure 5B), and Hungateiclostridiaceae (Figure 5C) were significantly increased after a dose of 3 Gy by 1–6-fold. In Hungateiclostridiaceae, this same dose also increased bacterial abundance in the colon. A different response was seen in the order Clostridiales. Here, the colon and feces were minimally impacted; however, a dose of 3 Gy produced a large increase in ileal abundance by 249-fold (Figure 5D).

Additional orders of the class Clostidia were analyzed, including Lachnospirales (Supplementary Figure S2A), Oscillospirales (Supplementary Figure S2B), Clostridia vadinBB60 group (Supplementary Figure S2C), and Peptococcales (Supplementary Figure S2D). All were significantly more abundant in the colon and feces (*p* ≤ 0.0001). However, Clostridia vadinBB60 group and Peptococcales comprise less than 2% of the GIT. These orders were minimally impacted by radiation exposure, with only a slight decrease in Oscillospirales in the ileum at 3 Gy and a slight increase in Clostridia vadinBB60 group in the feces following a dose of 0.25 Gy.

#### 2.2.3. Radiation Altered the Distribution of Bacteroidetes in the GIT

**Figure 6:**
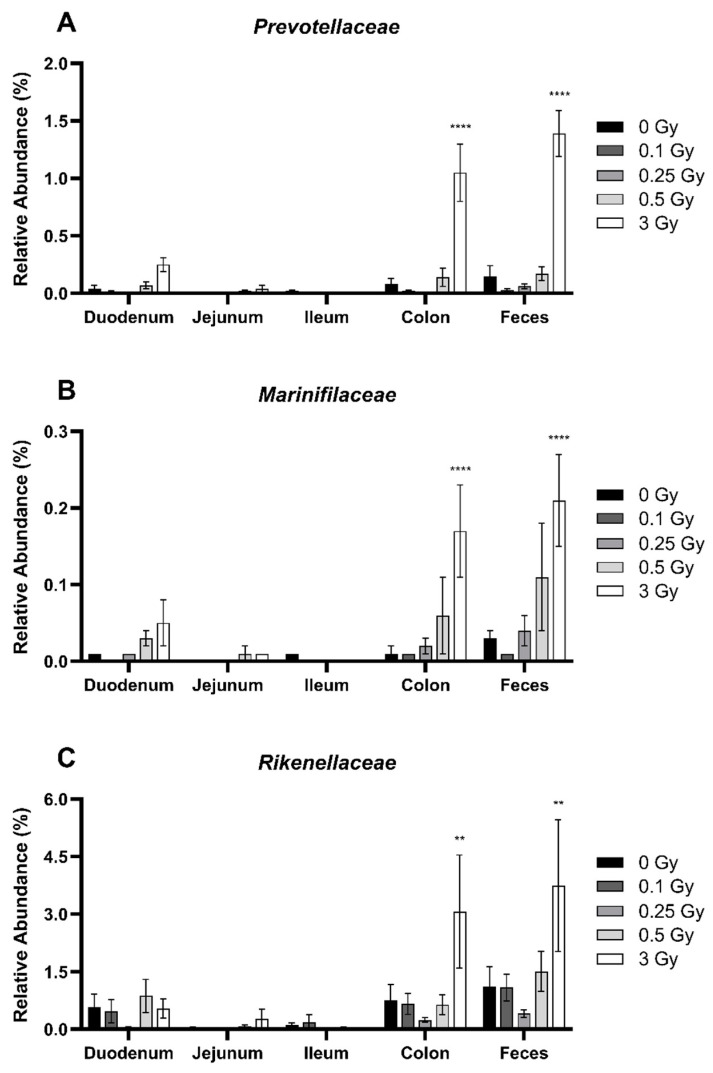
Relative abundance of the Bacteroidetes family members *Prevotellaceae* (**A**), *Marinifilaceae* (**B**), and *Rikenellaceae* (**C**) in the GIT and feces of irradiated mice. Relative bacterial abundance (% of total) was quantified using 16S metagenomics sequencing 48 h post radiation exposure. Bars represent the mean ± SEM across replicate animals (*n* = 5–12 per treatment). Data were analyzed using a two-way ANOVA followed by Tukey’s post hoc test. Symbols indicate statistical significance compared to 0 Gy control within each GIT segment: ** *p* ≤ 0.01, **** *p* ≤ 0.0001.

Similar to Firmicutes, the distribution of bacteria within the phyla Bacteroidetes was impacted by radiation despite no impact on the overall abundance. Family members *Prevotellaceae* (Figure 6A), *Marinifilaceae* (Figure 6B), and *Rikenellaceae* (Figure 6C) were present in low abundance, each comprising less than 1.5% of the GIT of sham-irradiated mice. However, these families were significantly more abundant in the GIT of mice following a dose of 3 Gy, specifically in the colon and feces. Compared to controls, *Prevotellaceae* was increased 13.9-fold in the colon and 9.4-fold in the feces. Similarly, *Marinifilaceae* increased by 12.0-fold in the colon and 8.0-fold in the feces. Finally, *Rikenellaceae* rose 4.1-fold in the colon and 3.4-fold in the feces. Of note, the magnitude of change was similar in both the colon and feces, suggesting that the sampling of the feces is a good indicator of the distribution of Bacterioidetes family members within the colon.

Additional members of the Bacteroidetes phyla *Bacteroidaceae* (Supplementary Figure S3A) and *Muribaculaceae* (Supplementary Figure S3B) were also impacted by exposure to ionizing radiation. However, this was not the case for the family *Tannerellaceae* (Supplementary Figure S3C). Compared to control mice, the relative abundance of *Bacteroidaceae* decreased by 2.8-fold in the feces, whereas the quantity of *Muribaculaceae* increased 6.3-fold in the ileum, both at a dose of 3 Gy.

#### 2.2.4. Radiation Altered the Distribution of Select Members of Proteobacteria and Deferribacterota Phyla in the GIT

**Figure 7:**
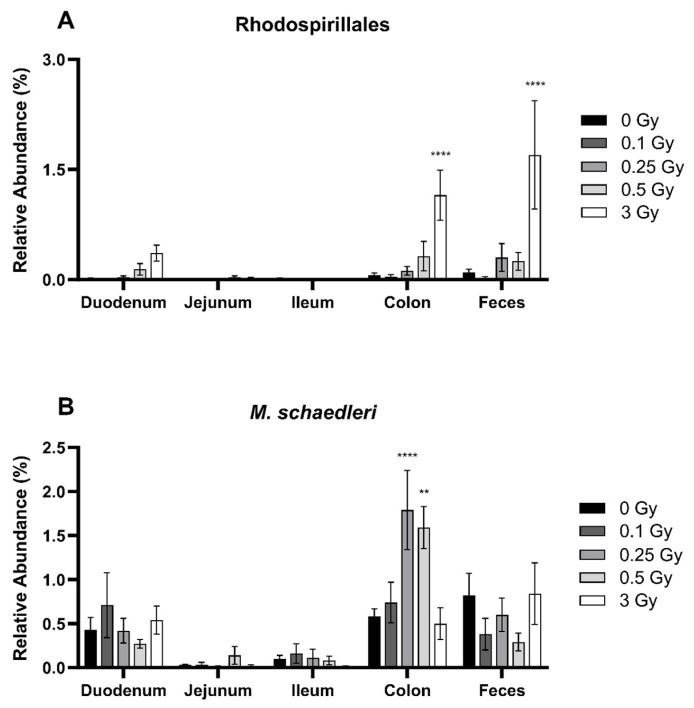
Relative abundance of the bacteria order Rhodospirillales of the Proteobacteria phyla (**A**), and species *Mucispirillum schaedleri* of the Deferribacterota phyla (**B**) in the GIT and feces of irradiated mice. Relative bacterial abundance (% of total) was quantified using 16S metagenomics sequencing 48 h post radiation exposure. Bars represent the mean ± SEM across replicate animals (*n* = 5–12 per treatment). Data were analyzed using a two-way ANOVA followed by Tukey’s post hoc test. Symbols indicate statistical significance compared to 0 Gy control within each GIT segment: ** *p* ≤ 0.01, **** *p* ≤ 0.0001.

Despite their low abundance of less than 2% in the GIT, radiation impacted the relative abundance of the bacterial order Rhodospirillales of the Proteobacteria phyla (Figure 7A) and species *Mucispirillum schaedleri* of the Deferribacterota phyla (Figure 7B). Rhodospirillales was significantly increased following a dose of 3 Gy in the colon and feces by 19.2- and 16.6-fold, respectively. *Mucispirillum schaedleri* was also significantly increased in the colon. Compared to sham-irradiated mice, the relative abundance of *Mucispirillum schaedleri* increased by 3.1- and 2.7-fold at doses of 0.25 and 0.5 Gy, respectively.

## 3. Discussion

**Figure 8:**
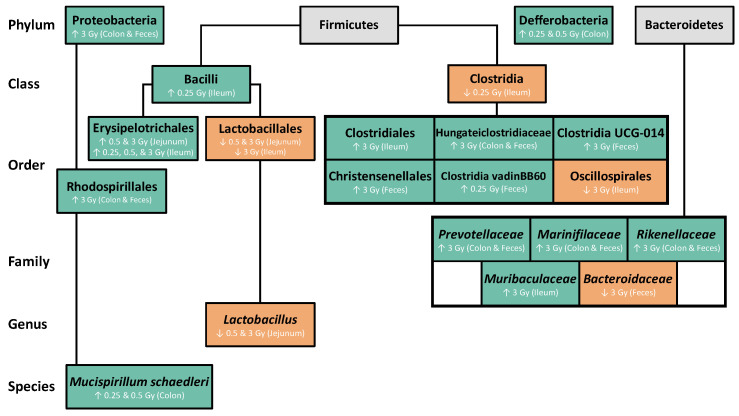
Summary of radiation-induced GIT microbial changes identified in this study. Green boxes (up arrows) and yellow boxes (down arrows) indicate significantly increased and decreased bacterial abundance 48 h post radiation exposure compared to sham, respectively. Grey boxes indicate no significant change. Details pertaining to the GIT section and radiation dose are provided for results with significance.

The goal of this study was to investigate short-term radiation effects on the GIT, both structurally and in the composition of the gut microbiome. C57BL/6 mice were exposed to X-rays from 0.1–3 Gy and the GIT was analyzed 48 h post irradiation. Based on the H&E staining of the ileum, there were no significant changes in villi height at any dose. However, crypt depth and goblet cell density were reduced, while goblet cell area was increased. In addition, we identified dose- and tissue-dependent shifts in bacterial abundance following radiation exposure, with changes observed as low as 0.25 Gy. While there were no significant changes in the overall abundance of Firmicutes or Bacteroidetes, the microbial distribution within each of these phyla was significantly impacted. Specifically, the relative abundance of Erysipelotrichales, Lactobacillales, Clostridiales, Oscillospirales, *Lactobacillus*, and *Muribaculaceae* was altered within the small bowels, while Clostridia UCG-014, Christensenellales, Hungateiclostridiaceae, Rhodospirillales, Clostridia vadinBB60 group, *Prevotellaceae*, *Marinifilaceae, Rikenellaceae*, and *Bacteroidaceae* were increased within the large bowels and feces. Figure 8 summarizes the key radiation-induced GIT microbial changes identified in this study.

It is well known that high-dose ionizing radiation can negatively impact the GIT, resulting in symptoms of acute radiation syndrome. These include the destruction of highly radiosensitive intestinal stem cells, shortening of crypts/villi, and increased permeability [3]. Previous studies on radiation-induced intestinal damage have primarily focused on longer term effects, over the span of days to weeks. The aim of the current study was to identify short-term acute changes in mice following low and high dose exposures. Our histological data focused specifically on radiation-induced changes to the ileum, which is the most common GIT segment that has been examined across previous studies [23,24,25,26]. We report significant changes in various parameters across the intestinal crypts, but not in villi height (Figure 1). Gupta et al. [27] also found no significant differences to the structure of the villi 24 h following a full body dose of 7 Gy; however, after 5 days, the length of the villi was significantly decreased. Similarly, Livanova et al. [28] found no impact on villus height in the jejunum 3 days following a 2 Gy exposure, but crypt depth was significantly affected. Multiple other studies have shown changes in crypt/villi size at timepoints greater than 3 days after exposure [23,28,29,30,31], albeit mainly at doses in excess of 10 Gy. Taken together, this suggests that the intestinal crypts respond more rapidly to high-dose irradiation, while the villi have a longer latency period for effects to manifest.

We also observed a much more significant response in goblet cell density compared to crypt depth. A dose of 3 Gy reduced the number of goblet cells by 37% compared to only a 16% decrease in crypt depth (Figure 1). This suggests that goblet cells, which comprise 8–10% of crypt cells [32], are more acutely sensitive to radiation compared to other enterocytes. Goblet cell destruction could also be seen as a precursor to overall crypt/villi damage given their physiological role of mucus secretion which helps to protect the GIT mucosa [33,34,35]. A reduction in goblet cell density, and a subsequent decrease in mucus production, could be a contributing factor to eventual crypt/villi shortening.

The largest radiation impact on the intestinal epithelium appeared to be in goblet cell size, demonstrated by the 66% increase compared to sham mice (Figure 1). To the best of our knowledge, this parameter has not been studied or reported on by others. However, Jang et al. [31] exposed mice to a single dose of 13.5 Gy targeted to the abdomen and after 6 days reported significant crypt destruction, with results indicating that irradiation caused goblet cell malformation and decreased the amount of mucin secreted within the small intestine. It is known that mucin is secreted through exocytosis and its production is increased in response to stressors [36]. Along with these physiological concepts, and the decreased mucin secretion seen by Jang et al. [31], our results propose that radiation exposure may impair these mechanisms, leading to larger goblet cells as a result of mucin being built up within the cells. Goblet cell response to intestinal injuries is poorly defined, and there is evidence to suggest that the functions of goblet cells are altered both directly by the gut microbiome and in response to cellular contact with bioactive factors following their exposure to bacteria [37].

Along with histological changes, we investigated radiation effects on the gut microbiome. The gut microbiomes of humans and mice have a reported 90% similarity at the level of the phyla and 89% similarity at the level of the genera [38]. In alignment with the typical composition, our results identified Firmicutes and Bacteroidetes as the dominant phyla within the GIT of mice, where Firmicutes comprised 80–99% of the microbiome depending on dose and region. Within the HGM specifically, Firmicutes and Bacteroidetes comprise roughly 80% at the level of the phyla [3,4,11]. An analysis of the gut microbiome of male C57BL/6 mice by Lkhagva et al. [9] revealed that the abundance of Firmicutes gradually decreased from the stomach to the feces, while, in contrast, the abundance of Bacteroidetes gradually increased throughout the GIT. Both of these findings align with our data (Figure 2). The environment of the lower GIT lacks oxygen, and, as such, anaerobic bacteria like those in the Bacteroidetes phylum are seen in higher abundance in the colon and feces [39]. We identified two other phyla, Proteobacteria and Defferobacteria, within all GIT regions, but each represented less than 1% abundance in sham-irradiated mice. Lkhagva et al. [9] also identified a Defferobacteria abundance of less than 1% in male C57BL/6 mice but found a slightly higher abundance of Proteobacteria of approximately 8%. Slight differences in age, rearing conditions, and diet could account for these discrepancies.

Following radiation exposure, our results showed no significant changes in alpha diversity and no alterations in the ratios of the dominant phyla. In agreement with our results, Segers et al. [23] found no significant alterations in the Shannon index following 16S metagenomic sequencing on the fecal samples of mice exposed to abdominal doses of 12 Gy and sacrificed 1, 3, or 7 days post irradiation. In contrast, other authors have reported significantly lower Shannon indices in irradiated mice compared to healthy controls [18,29]. For example, Zhang et al. [29] found a lower alpha diversity in mice 3 days after abdominal irradiation with 12 Gy. Similarly, Liu et al. [18] found that the Shannon index decreased significantly in fecal samples from mice exposed to low-dose gamma rays at a total dose of 0.5 Gy delivered in one or more fractions.

Looking within each phylum, we identified multiple shifts in the relative abundance of bacteria within specific classes, orders, and families. Comparably, few studies have investigated radiation effects at these taxonomic ranks. Of note, in agreement with our results, Zhang et al. [29] found that following 12 Gy irradiation, the abundance of the Bacteroidetes family *Prevotellaceae* was significantly altered. However, as opposed to our results, they found that the relative abundance was reduced, whereas our 3 Gy dose significantly increased *Prevotellaceae* (Figure 6A). Following 10 fractions of 0.05 Gy (total dose 0.25 Gy), Liu et al. [18] found that Clostridiales was increased, similar to what we identified within the ilium following exposures of 3 Gy (Figure 5D). Conversely, they also showed that the class Clostridia was increased after irradiation, whereas our results indicated a decrease in the ileum at 0.25 Gy (Figure 3B). Throughout these lower taxonomic ranks, our results indicated alterations in microbes such as Clostridia, Lactobacillales, *Prevotellaceae*, and *Lactobacillus* (Figure 8). These microbes have all been shown to be markers of inflammation [33,40]. Additionally, the genus *Lactobacillus* is known to protect the intestinal barrier from infection through various mechanisms [41]. Therefore, a decrease in *Lactobacillus,* such as the one we saw (Figure 4C), could lead to a decline in GI health through a cascade of events caused by the loss of these beneficial species.

Taken together, our results indicate that there is more to be seen beyond the level of the phylum which is where many other studies stop, and therefore fall short, regarding the amount of information that can be collected regarding microbial identity. We see that there can be changes deeper within the taxonomic ranks that need to be considered in future studies. Many previous studies highlight the effects of much higher doses of radiation on the GIT and the microbiome. However, it is important to understand the potential impacts of lower doses. Our study provides new information to help fill this knowledge gap within the mid- to low-dose range.

Our data highlighted radiation-induced alterations in the structure of the GIT as well as the gut microbiome. It is possible that these effects are not the direct result of radiation exposure to the GIT but rather are an indirect response to some other radiation side effect. However, we did not observe any noticeable changes in the mice between the time of irradiation and sacrifice. In particular, we did not see any alterations in food/water intake or bowel function, which could impact microbiome composition. Exposure to ionizing radiation has been shown to elicit effects such as fluctuation in food intake and loss of appetite; however, these generally become apparent more that 48 h following irradiation [4]. In addition to this, it is known that the GIT is a highly radiosensitive structure that is impacted quickly after radiation exposure. Taken together, we believe that the effects we observed were the direct result of radiation interactions with the GIT.

The intestinal mucosa and the microbiome are heavily interconnected and provide bidirectional signals to maintain optimal gut health. On their own, bacteria are known to be highly resistant to ionizing radiation exposure [42,43,44]. Therefore, our highest dose of 3 Gy would be unlikely to directly alter microbial survival. Instead, it is likely that radiation exposure induced changes in the GIT microenvironment, which indirectly resulted in bacterial dysbiosis. We identified a significant reduction in goblet cell density but an increase in goblet cell size. Goblet cells are responsible for mucus secretion along the GIT, which forms a barrier covering the epithelium and allows for the separation of the gut microbiome from the mucosa [34,35]. When the integrity of the mucus layer is compromised, microbes are given the chance to interact with the epithelium, leading to a cascade of events such as the degeneration of tight-junction proteins, increased permeability, and inflammatory responses [21,45]. Another role of the mucus layer is to support the growth of a microbial community known as the mucus-associated microbiota [33]. The population of these microbes is largely reliant on the health of the mucus layer, and, as such, they are susceptible to being affected by an acute decrease in goblet cells [20,33]. As a symbol of their symbiotic relationship, the formation of the mucus layer itself is dependent on these microbes, and low diversity of the mucosal microbiome has been reported following irradiation [4,33]. The mucosa has been shown to harbour significant amounts of bacteria from the Firmicutes and Proteobacteria phyla, which has been shown to contribute to the presence of a well-defined mucus layer [33]. Our analysis indicated an increase in Proteobacteria in the colon and feces following exposures to 3 Gy. An increase in this phylum has been reported as a common finding in the fecal samples of rodents following radiation exposure [4]. *Lactobacillus* species have also been found to stimulate mucin secretion in the goblet cells of the human GIT [33]. In alignment with this, our data show a decrease in the relative abundance of *Lactobacillus* within the small intestine following 3 Gy.

Damage to the GIT is a common side effect in patients receiving radiotherapy for abdominal or pelvic malignancies. Previous studies have shown that large, accumulated doses, across multiple radiotherapy fractions, can alter the microbiome composition and damage the gut mucosa. For example, Mitra et al. [46] found a decrease in microbiome diversity in chemoradiation patients up to 5 weeks into treatments. However, the severity of these side effects can vary greatly between individuals depending on factors such as age, sex, health, and genetic predisposition. Therefore, early predictors of GIT toxicities could be vital to the effective management of radiation-induced bowel damage in susceptible individuals. For this reason, we wanted to investigate short-term changes to the GIT that could manifest within 48 h of a single radiotherapy fraction. Our highest dose of 3 Gy was selected to represent an average dose fraction to the GIT across conventional and hypofractionated therapies.

It has been suggested that the fecal microbiome is an inaccurate marker of the overall gut microbiome [9]. However, for human applications, fecal analysis can be an important non-invasive tool for profiling gut health. In fact, our data suggest that the feces can be a reliable indicator of changes to the colon microbiome when looking at Bacteroidetes family members (Figure 6). Previous studies have used human feces as an indicator for radiation therapy-induced side effects and have been successful in determining the dysbiosis of specific microbes linked to the development of adverse outcomes in patients, including the genus *Clostridium* XI and XVIII and the families *Eubacteriaceae, Fusobacteriaceae, Streptococcaceae, Veillonellaceae, Enterococcaceae*, and *Lactobacillales* [13,14]. Within our study, we identified various microbial changes in the feces of mice following 3 Gy. In particular, we showed a large increase in the phyla Proteobacteria (Figure 2), as well as various orders/families of Firmicutes (Figure 5) and Bacteroidetes (Figure 6). Future studies should profile these specific microbes in fecal samples from radiotherapy patients.

Monitoring changes within the gut microbiome is a valuable tool that may be used as a biomarker of early stages of bowel damage. Tracking changes in the abundance of these microbes could play an important role in the implementation of mitigation strategies for radiation therapy-induced bowel injury. One example of these treatments is fecal transplantation, which has recently gained popularity for its success in treating patients with recurrent *Clostridium difficil* infections through the restoration of normal microbial diversity [47]. This mitigation strategy of transferring feces from a healthy microbiome has been shown to mitigate various characteristics of disease that are linked to microbial dysbiosis [47]. Fecal transplantation has also shown high levels of success in the mediation of various other conditions, including radiation therapy-induced dysbiosis [48,49].

In conclusion, this study provides insight into how ionizing radiation exposure can induce acute alterations across various taxonomic ranks in the microbiomes of mice. Overall, these data could lead to potential biomarkers for acute monitoring in radiotherapy patients or targets for mitigation strategies, leading to adjuvant therapies for the management of side effects and improved disease control. The gut microbiome is an integral part of maintaining GIT homeostasis, which can greatly impact the body in a multisystemic manner, and its role in human health only continues to grow. With various studies pointing to its role in the management of cancer as well as treatment-induced side effects, it is important to understand how ionizing radiation exposure can impact the gut microbiome. Cancer therapies are notoriously linked to high rates of morbidity and a reduced quality of life among patients. The GIT is one of the most sensitive systems to cancer therapies and being able to protect it from these adverse effects remains an unmet need in patient care.

## 4. Materials and Methods

### 4.1. Animal Handling

A cohort of 40 male C57BL/6 mice were purchased from Charles River Laboratories (Charles River Laboratories, Saint-Constant, QC, Canada) and were between 7 and 12 weeks of age. All mice were housed at the Laurentian University Animal Facility under a 12/12 h light/dark cycle and their feed was supplied ad libitum. The animals underwent a minimum 4-week acclimation period prior to irradiation. All animal protocols were approved by the Laurentian University Animal Care Committee in accordance with guidelines from the Canadian Council on Animal Care.

### 4.2. Irradiations

At 16 weeks of age, mice were exposed to various doses of ionizing radiation via an in-house X-Rad 320 irradiation cabinet (Precision X-ray, Madison, CT, USA). The machine was operated at 320 kV with a 2 mm Al filter. Mice were exposed to a full body single acute dose of 0.1, 0.25, 0.5, or 3 Gy. For the 0.1 and 0.25 Gy doses, the tube current was set to 0.5 mA, resulting in a dose rate of approximately 0.045 Gy/min. For the 0.5 and 3 Gy doses, the tube current was set to 12.5 mA, resulting in a dose rate of approximately 1.5 Gy/min. To adequately immobilize the mice throughout treatment, they were placed inside of a pie cage which was placed at a distance of 60 cm from the source. Sham-irradiated mice were placed in the pie cage within the X-ray unit without turning on the machine. Once irradiated, the mice were transported back to the animal facility for 48 h before tissue collection. During the 48 h, mice were monitored for any changes to their typical function such as appetite, water intake, bowel habits, and behavior. Prior to experimental treatments, absorbed doses were verified using LiF thermoluminescent dosimeters (Mirion Technologies, Atlanta, GA, USA), which were placed at various locations within the bodies of deceased mice.

### 4.3. Tissue Collection

Mice were anaesthetized by the administration of isoflurane gas and euthanized via cervical dislocation. Following a cardiac puncture to drain the blood, the abdomen was opened, and the full GIT was removed. For the small bowel, 3–4 cm sections of the duodenum, jejunum, and ilium were cut and flushed using sterile deionized water to collect luminal contents. Prior to the sectioning of the colon, the feces were removed from the tissue to be used for microbiome analysis. The tissue samples were then cut into smaller 1 cm pieces and stored in formaldehyde (for histological analysis), or were flash-frozen in liquid nitrogen with subsequent storage at −80 °C (for microbiome analysis).

### 4.4. Histological Analysis

Based on the previous literature [23,24,25,26], the ileum is a common GIT segment which is analyzed post radiation exposure. Collected sections of the ileum were hematoxylin and eosin (H&E)-stained to determine the histological effects of irradiation on the GIT. Following harvest from the mice, tissues were post-fixed in 10% buffered formalin for 24 h and stored in 70% ethanol where they were then transported to the Pathology Research Program of the University Health Network (UHN) in Toronto, ON, Canada. At the facility, the samples of wet-fixed ileum were processed, embedded on edge, cut into 4–5 µm thick cross-sections, and H&E-stained.

Once the stained tissue samples were received back from the UHN, a Cytation 5 cell imaging multimode reader (Agilent (formerly BioTek), Winooski, VT, USA) was used to image the H&E-stained ileal tissues. The ImageJ analysis program (v1.54f, National Institutes of Health, Bethesda, MD, USA) was then used to quantify villi height, crypt depth, and goblet cell number/size [50]. To score villi height and crypt depth, a straight-line tool was used to perform manual measurements on images taken at 10×. Villus height was defined as the distance between the crypt/villus junction and the tip of the corresponding villus, whereas crypt depth was defined as the length between the crypt/villus junction and the base of the crypt [51]. Goblet cells were scored at 40×. The number of goblet cells was manually counted, and the area was measured by drawing around the circumference of the cell. An average of 24 villi and 103 crypts were scored per animal.

### 4.5. 16S Metagenomics Microbiome Analysis

Total genomic DNA was extracted from flash-frozen GIT regions and fecal samples using the Quick-DNA™ Fecal/Soil Microbe Miniprep Kit (D6010, Zymo Research, Irvine, CA, USA) according to the manufacturer’s instructions. The purified DNA samples were analyzed using the ND-1000 Nanodrop spectrophotometer to measure the absorbance ratio at 260/280 nm and 260/230 nm in order to assess DNA purity and concentration. DNA samples below a 260/280 absorbance ratio of 1.8 were excluded from analysis. The DNA samples were sent to the McMaster Genomics Facility (McMaster University, Hamilton, ON, Canada) for 16S metagenomics library preparation and sequencing. Briefly, a two-stage PCR amplification protocol was used for generating the metagenomics library. The first PCR stage amplified the V3 and V4 regions of the 16S ribosomal RNA gene (~460 base pairs) for each sample using limited PCR cycle numbers. The PCR amplicon was isolated using bead purification. The second-stage PCR resulted in amplicons appended with a unique sample barcode for each sample, in addition to Illumina’s overhang adapter sequences. The amplicons were bead-purified and pooled at equimolar concentration. The pooled library was sequenced on a MiSeq (Illumina, San Diego, CA, USA) instrument using a Version 3 reagent kit that enabled 300 base-pair paired-end reads for a total of 25 million reads. This resulted in approximately 100,000 reads per sample, commonly recognized as sufficient for metagenomic analysis.

The sequencing data were processed using the DADA2 pipeline, as described previously [52], in order to generate read counts for each sample across the different taxonomic categories. Percent relative abundance data were generated for each taxonomic line item using the total read count for each sample. Overall, this workflow is referred to as Operational Taxonomic Unit (OTU) clustering.

Changes in gut microbiome composition were also measured in the different GIT regions using alpha diversity to determine the diversity within single samples. Specifically, the Shannon–Wiener (*H′*) diversity index was chosen as it takes into account both the number of species in a sample (evenness) and their relative abundance (richness) [53]. The Shannon index measures the entropy of species and is delineated by the equation

$$H^{\prime }=-\sum_{i=1}^{s}\left(P_{i}\ln P_{i}\right)$$

with *P_i_* as the proportion of individuals belonging to the *i*th species in the dataset [53]. The Shannon species diversity results were populated through Illumina’s BaseSpace platform.

### 4.6. Statistical Analysis

Statistical analyses were performed with GraphPad Prism (Version 8.4.3, GraphPad Software Inc., San Diego, CA, USA). For histological data, a one-way analysis of variance (ANOVA) followed by Tukey’s post hoc test was performed to analyze the effects of radiation dose on villi length, crypt depth, goblet cell size, and goblet cell density. For 16S metagenomic sequencing outputs, a two-way ANOVA followed by Tukey’s post hoc test was used to determine statistical significance for Shannon indices and bacterial composition, with the radiation dose and GIT segment as the two independent variables. *p*-values ≤ 0.05 were considered statistically significant.

## Data Availability

The raw data supporting the conclusions of this article will be made available by the authors on request.

## Supplementary Materials

The following supporting information can be downloaded at https://www.mdpi.com/article/10.3390/ijms25063339/s1.
