# Supplementary material for: Acute Impacts of Ionizing Radiation Exposure on the Gastrointestinal Tract and Gut Microbiome in Mice
Source: Int J Mol Sci. 2024 Mar 15;25(6):3339. doi: 10.3390/ijms25063339 (PMC10970505; doi:10.3390/ijms25063339)
Supplement: Supplementary file 1 [file ijms-25-03339-s001.zip › ijms-2880715-supplementary.pdf]

Supplementary Figures

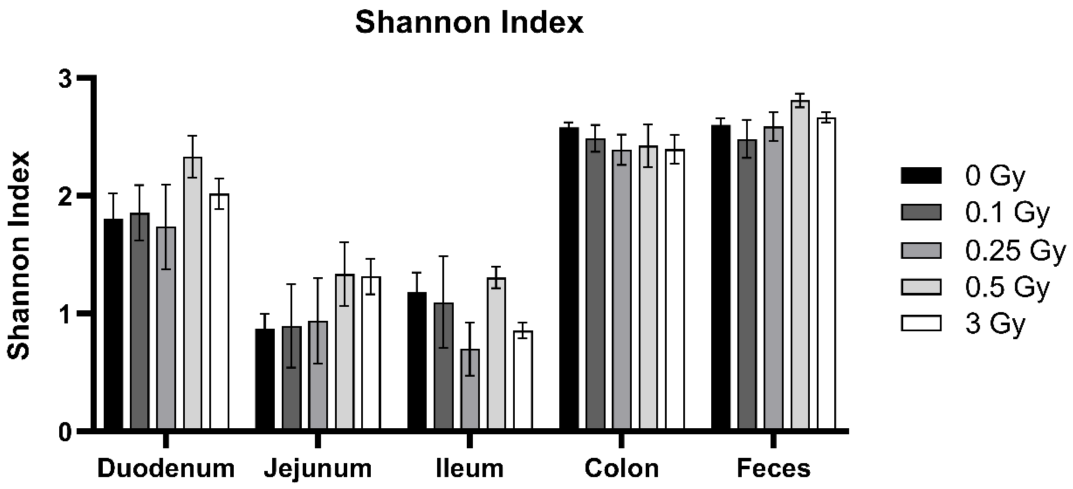

Supplementary Figure S1. Shannon-Wiener alpha diversity index in the GIT and feces of irradiated mice. Bars represent the mean ± SEM across replicate animals (n = 5–12 per treatment). Data were analyzed using a one-way ANOVA followed by Tukey's post-hoc test.

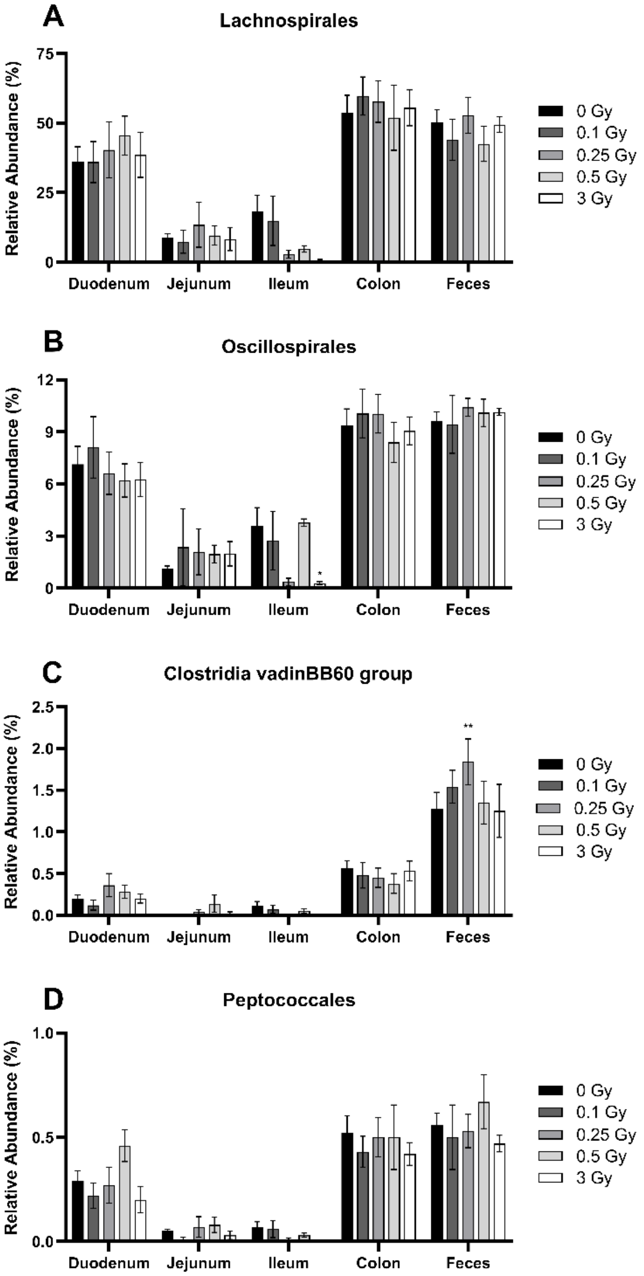

Supplementary Figure S2. Relative abundance of the bacteria order Lachnospirales (A), Oscillospirales (B), Clostridia vadinBB60 group (C), and Peptococcales (D) in the GIT and feces of irradiated mice. These microbes belong to the bacterial class Clostridia, under the phyla Firmicutes. Relative bacterial abundance (% of total) was quantified using 16S metagenomics sequencing 48 hours post radiation exposure. Bars represent the mean ± SEM across replicate animals (n = 5–12 per treatment). Data were analyzed using a two-way ANOVA followed by Tukey's post-hoc test. Symbols indicate statistical significance compared to 0 Gy control within each GIT segment: * p≤0.05, ** p≤0.01.

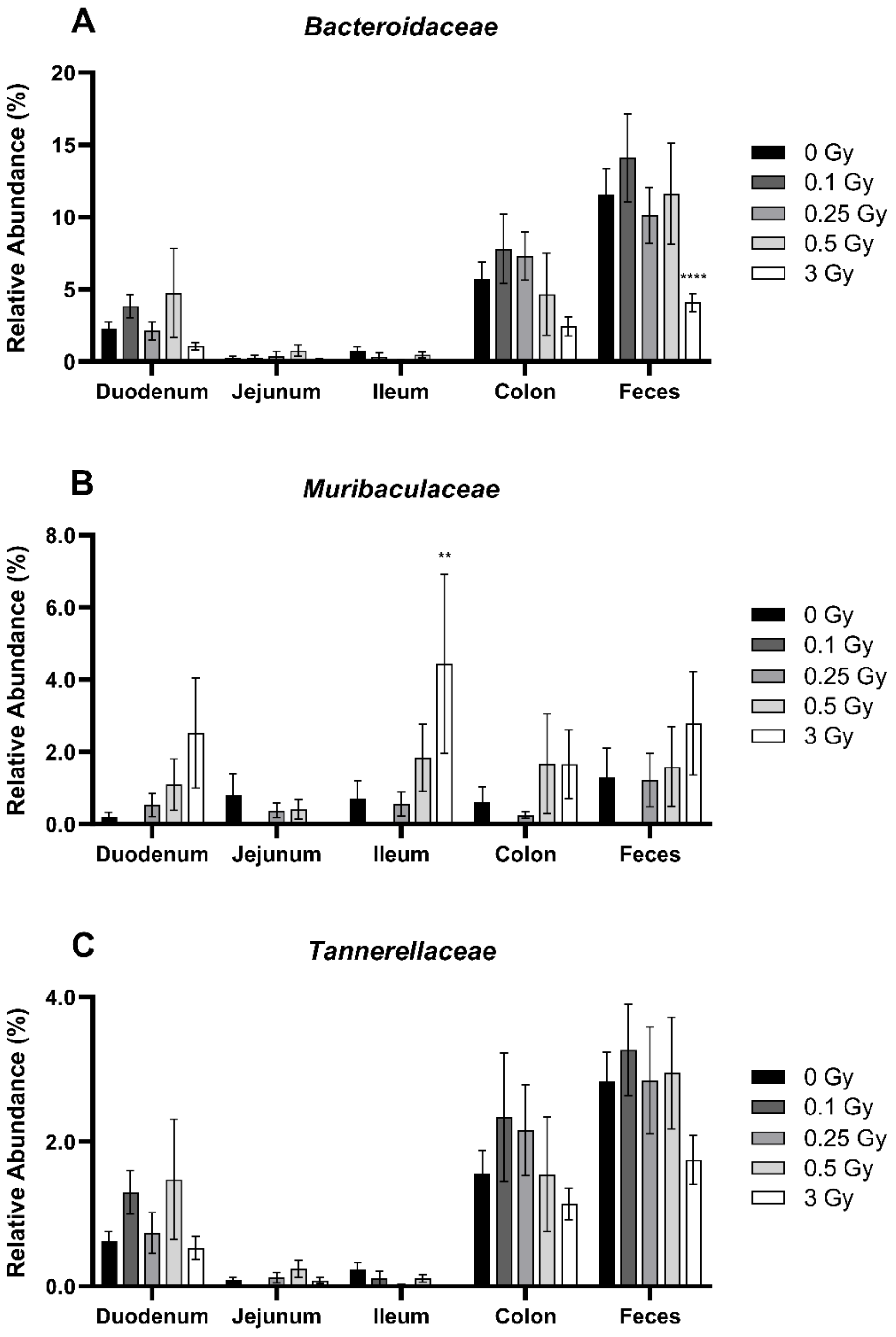

Supplementary Figure S3. Relative abundance of Bacteroidetes family members *Bacteroidaceae* (A), *Muribaculaceae* (B), and *Tannerellaceae* (C) in the GIT and feces of irradiated mice. Relative bacterial abundance (% of total) was quantified using 16S metagenomics sequencing 48 hours post radiation exposure. Bars represent the mean ± SEM across replicate animals (n = 5–12 per treatment). Data were analyzed using a two-way ANOVA followed by Tukey's post-hoc test. Symbols indicate statistical significance compared to 0 Gy control within each GIT segment: ** $p \leq 0.01$, **** $p \leq 0.0001$.
